# Allergic Contact Dermatitis From Dorzolamide Without Cross‐Reactivity to Brinzolamide

**DOI:** 10.1111/cod.70151

**Published:** 2026-04-06

**Authors:** Elias Ghozli, Camille Leleu, Louis Arnould, Louise Collin, Evelyne Collet

**Affiliations:** ^1^ Department of Dermatology Contact Allergy Unit, François Mitterrand Hospital, Dijon University Hospital Dijon France; ^2^ Department of Ophthalmology François Mitterrand Hospital, Dijon University Hospital Dijon France

**Keywords:** allergic contact dermatitis, brinzolamide, carbonic anhydrase inhibitors, case report, cross‐reactivity, dorzolamide

Dorzolamide and brinzolamide are topical carbonic anhydrase inhibitors (CAIs) used to reduce intraocular pressure in glaucoma and treat macular oedema in retinitis pigmentosa. Although several cases of eyelid contact dermatitis have been reported [1, 2], all drugs within the CAI class are usually contraindicated due to their similar chemical structures and the potential risk of cross‐reactivity. We report a case of allergic contact dermatitis from dorzolamide, confirmed by patch testing, without cross‐reactivity to brinzolamide.

## 1 Case Report

A 58‐year‐old atopic male, followed for retinitis pigmentosa complicated by macular oedema since 2016, was treated with Trusopt eye drops (dorzolamide 20 mg/mL—Santen laboratory).

**FIGURE 1:**
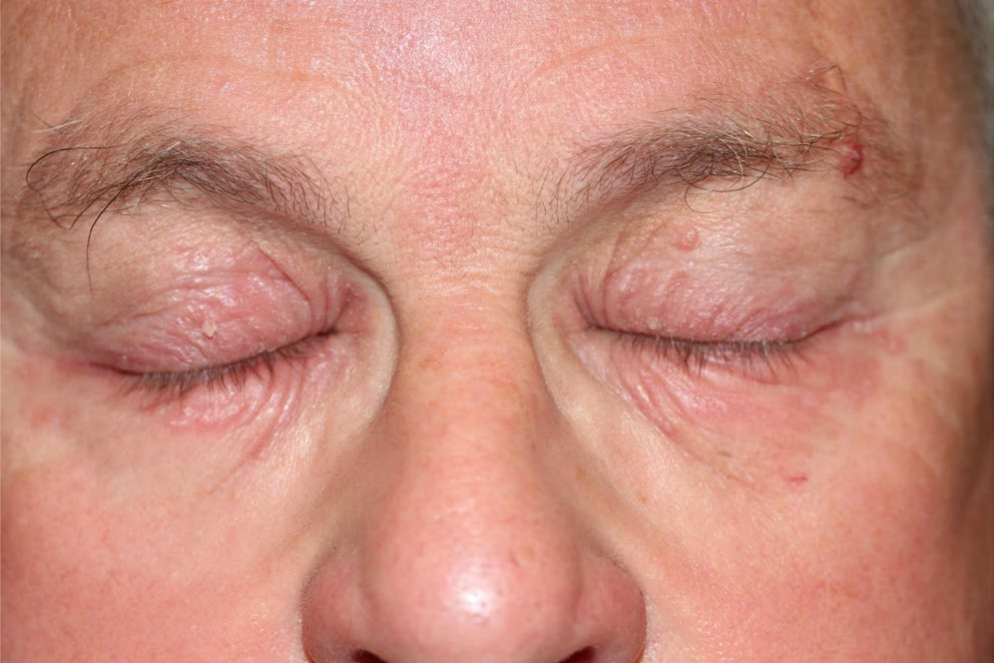
Eyelid eczema after the use of Trusopt eye drops (dorzolamide).

A few weeks after introduction of this eye drop, he developed chronic eyelid eczema and conjunctivitis (Figure 1). Treatment with Trusopt was discontinued and replaced by Azopt (brinzolamide 10 mg/mL—Novartis laboratory) with a regression of the allergic manifestations. Two months later, Azopt was discontinued because it was considered less effective on the macular edema, and Trusopt was reintroduced. The conjunctivitis and eyelid eczema rapidly recurred.

**FIGURE 2:**
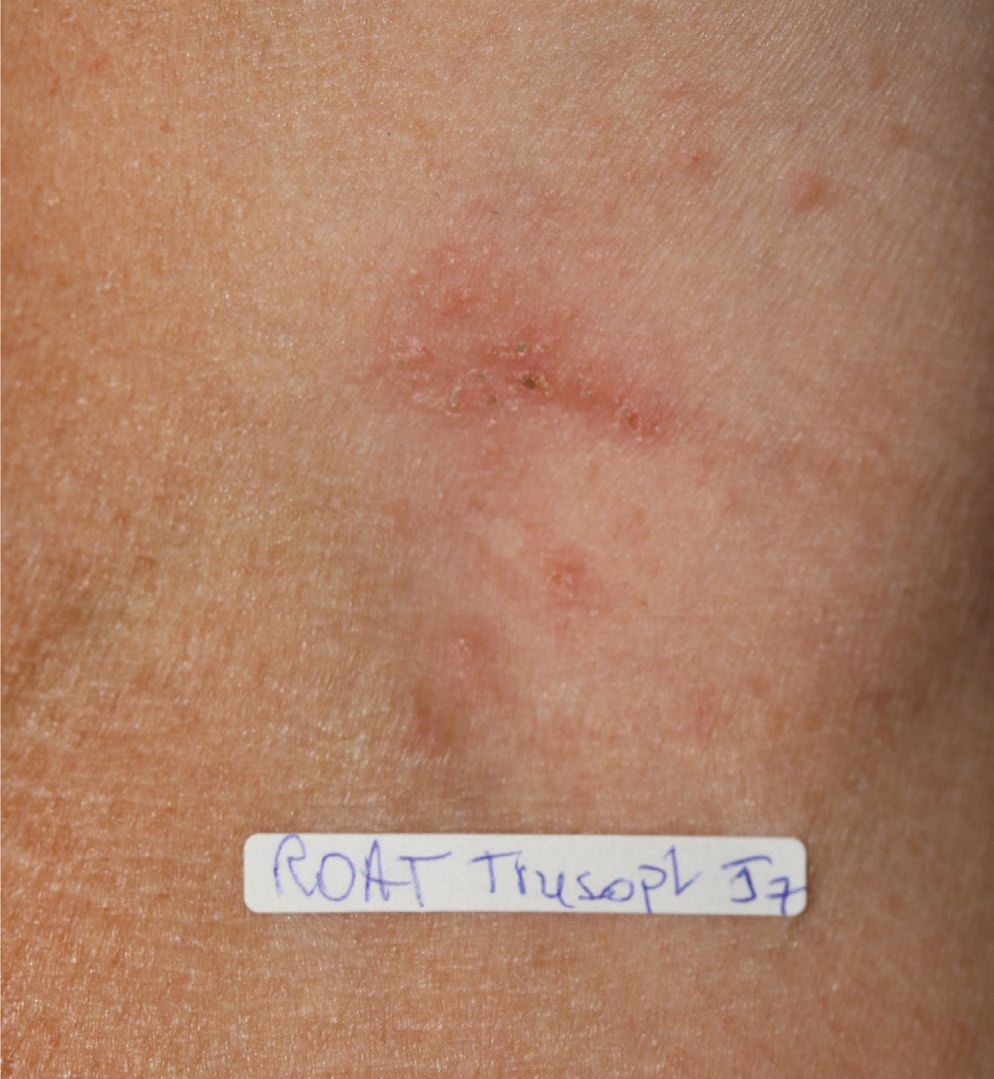
ROAT (Repeated Open Application Test) positive for Trusopt eye drops at D7.

The allergological investigation included patch tests (Chemotechnique Diagnostics, Vellinge, Sweden) with the two eye drops (Trusopt and Azopt) tested as is, read at D2 and D4 according to the ESCD (European Society of Contact Dermatitis) recommendations [3], and ROAT (Repeated Open Application Test). An acetazolamide patch test was also performed (Diamox, Cooper laboratory, powder for injection 30% pet). Trusopt and Azopt contain several common excipients, including benzalkonium chloride. Other allergenic excipients, such as EDTA and carbomer 974P, are found only in Azopt but not in Trusopt. The patch test for Trusopt eye drops was positive at D4 (++). The ROAT for Trusopt was also positive at D7 (Figure 2). The patch tests and ROAT for Azopt eye drops, as well as the patch tests for acetazolamide, were negative. Discontinuation of Trusopt and resumption of Azopt led to the patient's recovery.

## 2 Discussion

Several cases of allergic contact dermatitis of the eyelids with conjunctivitis from dorzolamide and one case from brinzolamide, confirmed by patch testing, have been reported [1, 2]. Co‐sensitizations have been described with benzalkonium chloride, latanoprost, and timolol [4]. Both molecules contain a sulfonamide group suspected in the occurrence of cross‐reactivity between these eye drops. It is therefore usual to contraindicate all CAIs in case of contact allergy despite controversial data in the literature [5].

Our observation shows the absence of cross‐reactivity in patch testing between brinzolamide and dorzolamide. The reintroduction of brinzolamide did not lead to a recurrence of eyelid eczema in our patient. The patch‐test was also negative for acetazolamide. This case confirms the first case recently reported by Otero‐Fernández et al. [6].

We report the second case investigated by patch testing and a reintroduction test showing the absence of cross‐reactivity between dorzolamide and brinzolamide.

## Author Contributions

**E. Ghozli:** writing – original draft.

## Consent

Written informed consent was obtained from the patient for the publication of clinical details and photographs.

## Conflicts of Interest

The authors declare no conflicts of interest.

## Data Availability

The data that support the findings of this study are available from the corresponding author upon reasonable request.
